# Supplementary material for: Effect of vascular simulation training on practice performance in residents: a retrospective cohort study
Source: BMJ Open. 2020 Sep 21;10(9):e037338. doi: 10.1136/bmjopen-2020-037338 (PMC7507846; doi:10.1136/bmjopen-2020-037338)
Supplement: Author's manuscript [file bmjopen-2020-037338.draft_revisions.pdf]

BMJ Open

BMJ Open is committed to open peer review. As part of this commitment we make the peer review history of every article we publish publicly available.

When an article is published we post the peer reviewers' comments and the authors' responses online. We also post the versions of the paper that were used during peer review. These are the versions that the peer review comments apply to.

The versions of the paper that follow are the versions that were submitted during the peer review process. They are not the versions of record or the final published versions. They should not be cited or distributed as the published version of this manuscript.

BMJ Open is an open access journal and the full, final, typeset and author-corrected version of record of the manuscript is available on our site with no access controls, subscription charges or pay-per-view fees (http://bmjopen.bmj.com).

If you have any questions on BMJ Open's open peer review process please email info.bmjopen@bmj.com

BMJ Open

Vascular simulation training promotes clinical skill and reduces radiation in residents

Journal:	*BMJ Open*
Manuscript ID	bmjopen-2020-037338
Article Type:	Original research
Date Submitted by the Author:	30-Jan-2020

Complete List of Authors:	Yang, Lin; First hoapital of Xi'an Jiaotong University, vascular surgery<br>Li, Yanzi; First hoapital of Xi'an Jiaotong University, Department of Medical Administration<br>Liu, Jianlin; First hoapital of Xi'an Jiaotong University, vascular surgery<br>Liu, Yamin; First hoapital of Xi'an Jiaotong University, international radiology

Keywords:	Vascular surgery < SURGERY, MEDICAL EDUCATION & TRAINING, Interventional radiology < RADIOLOGY & IMAGING, VASCULAR MEDICINE, VASCULAR SURGERY

SCHOLARONE™
Manuscripts

I, the Submitting Author has the right to grant and does grant on behalf of all authors of the Work (as defined in the below author licence), an exclusive licence and/or a non-exclusive licence for contributions from authors who are: i) UK Crown employees; ii) where BMJ has agreed a CC-BY licence shall apply, and/or iii) in accordance with the terms applicable for US Federal Government officers or employees acting as part of their official duties; on a worldwide, perpetual, irrevocable, royalty-free basis to BMJ Publishing Group Ltd ("BMJ") its licensees and where the relevant Journal is co-owned by BMJ to the co-owners of the Journal, to publish the Work in this journal and any other BMJ products and to exploit all rights, as set out in our licence.

The Submitting Author accepts and understands that any supply made under these terms is made by BMJ to the Submitting Author unless you are acting as an employee on behalf of your employer or a postgraduate student of an affiliated institution which is paying any applicable article publishing charge ("APC") for Open Access articles. Where the Submitting Author wishes to make the Work available on an Open Access basis (and intends to pay the relevant APC), the terms of reuse of such Open Access shall be governed by a Creative Commons licence – details of these licences and which Creative Commons licence will apply to this Work are set out in our licence referred to above.

Other than as permitted in any relevant BMJ Author's Self Archiving Policies, I confirm this Work has not been accepted for publication elsewhere, is not being considered for publication elsewhere and does not duplicate material already published. I confirm all authors consent to publication of this Work and authorise the granting of this licence.

For peer review only

Vascular simulation training promotes clinical skill and reduces radiation in residents

Lin Yang[1**], MD., Ph.D. Yanzi Li[2*], MD. Jianlin Liu[1], MD. Yamin Liu[3], MD

1. Department of Vascular Surgery, First Affiliated Hospital of Xi'an Jiaotong University, Xi'an, China
2. Department of Medical Administration, First Affiliated Hospital of Xi'an Jiaotong University, Xi'an, China
3. Department of Interventional Radiology, First Affiliated Hospital of Xi'an Jiaotong University, Xi'an, China

* Yanzi Li is the co-first author.

****Corresponding author:** LIN Yang. Department of Vascular Surgery, First Affiliated Hospital of Xi'an Jiaotong University, Xi'an, China. Tel.: +86 29 85324050. Fax: +86 29 85324050. *Email:* jdvascs@163.com

Running head: ST promote skill performance

For peer review only

Objective This study aims to investigate the teaching effect of vascular simulation training in rotating vascular residents. **Methods** A total of 95 vascular surgery residents were divided into a simulation training (ST) group and a conventional training (CT) group. The ST group received simulation training and conventional training, and the CT group only received conventional training. All data were collected, theoretical scores were assessed, and the technique parameters, complications and radiation damage of the procedures were analyzed. **Results** The mean scores (8.74±1.09 vs 8.13±1.31) and the rate of willingness for retraining (93.62% vs 79.17%) in residents were higher in the ST group than in the CT group (P<0.05). The success rate of arterial puncture was significantly higher in the ST group (78.72% vs 58.33%, P=0.03); however, the incidence of complications was similar between the two groups (P>0.05). The time of the puncture procedure was significantly lower (9.56± 5.24 min vs 12.15±6.87 min), and the comfort score of the patient (5.49±1.72 vs 4.71±1.57) was higher in the ST group than in the CT group (P<0.05). At the end of the assessment, the learning time for angiography (3.65±0.64 mon vs 4.07±0.77 mon) and the complete procedure time (33.81±10.11 min vs 41.32±12.56 min) were lower in the ST group than in the CT group (P<0.01). The fluo time for angiography (489.33±237.13s vs 631.47±243.65 s) and the cumulative air kerma (401.30±149.06 mGy vs 461.16±134.14 mGy) were significantly decreased in ST group (P<0.05). **Conclusion** The application of a vascular simulation system can significantly improve the clinical skills of residents and reduce the radiation damage from a single intervention procedure in patients.

Keywords: Vascular surgery; Teaching modes; Simulation training; Traditional

For peer review only - http://bmjopen.bmj.com/site/about/guidelines.xhtml

teaching

Article summary

Strengths and limitations of this study

- ◆ The simulation training could promote the mean scores of residents
- ◆ The simulation training could significantly improve the clinical skill of residents
- ◆ The simulation training could reduce the radiation damage
- ◆ This study was not a prospective randomized study, and simulation training was not used for every resident; thus, the conclusions of this study should be confirmed in the future.

For peer review only

For peer review only - http://bmjopen.bmj.com/site/about/guidelines.xhtml

INTRODUCTION

In recent years, with changes in the disease spectrum of Chinese patients, the incidence of peripheral arterial disease has increased significantly, which has also caused a severe economic and social burden [1]. Therefore, it is becoming increasingly important to strengthen general and specialized vascular disease skills education in the training of medical students and residents [2]. Vascular diseases involve multiple disciplines, such as general surgery, cardiology, endocrinology and interventional radiation, which also results in clinical training in vascular surgery being highly complex, with integration and multidisciplinary characteristics [3]. In the past decade, the practical skills training of resident has been mainly carried out through conventional teaching (CT) modes. From theoretical knowledge to practical procedures, residents lack sufficient practical procedures with simulation training; therefore, the true teaching effect has not been ideal. Three-dimensional vascular simulator systems (Angiomentor system, Simbionix, Ltd., Cleveland, OH) use digital simulation to quantify the vascular interventional procedures of the cardiovascular, peripheral and cerebrovascular systems. Students and residents can use the system to select cases for simulation training; ultimately, the simulation training results are scored according to the operating steps of the system. This simulation training can promote the mastery of vascular procedure skills in residents and students [4-6].

The simulation system may be used as an educational tool for novice students and residents, as it provides an opportunity to perform endovascular procedures in a safe

For peer review only - http://bmjopen.bmj.com/site/about/guidelines.xhtml

environment. However, due to the late entry of simulation systems in China, there have been no reports of the use of 3-dimensional vascular simulation systems in clinical practice teaching in the area of vascular surgery. The aim of this study was to assess the effect of simulation-based training on improving the technical performance and subsequent clinical procedures of residents in vascular surgery.

METHODS

Study procedures

A total of 95 vascular resident trainees at the First Affiliated Hospital of Xi'an Jiaotong University were recruited in this study from Jan 2015 to Dec 2018, and all residents needed to complete 6 months of endovascular training in vascular surgery. Thereafter, 47 vascular residents received simulation-based vascular training (ST group) for two weeks, and then they completed the last clinical training. The other 48 residents only completed conventional clinical training (CT group) without the simulation course. A survey was administered to determine the demographics, academic degree, specialty level, and previous work experiences that may have been relevant in terms of the residents' ability to learn vascular interventional skills. This study was approved by the institutional review board of the First Affiliated Hospital of Xi'an Jiaotong University (XJTU1AF2014LSK-112), all of the patients provided written informed consent.

Before the course was performed, the residents in the ST group received a standardized introduction to the endovascular simulator and performed a cerebrovascular angiography procedure. The 2-week curriculum consisted of theory teaching and 30-60 min lectures per day covering basic catheter-based interventions,

For peer review only - http://bmjopen.bmj.com/site/about/guidelines.xhtml

cerebrovascular disease, superficial femoral artery disease and renal artery disease. The residents received 1-hour mentored simulator sessions per day and practiced carotid, renal and superficial femoral artery interventions. This course was performed by a tutor. Then, residents needed to complete the primary endovascular procedure with direct instruction. During the entire training process, each resident was required to complete the simulation training for no less than 1 hour per day. The course concluded with a final cerebrovascular angiography procedure performed on the simulator. The residents in the CT group underwent the basic 2-week curriculum consisting of theory teaching and 30-60 min lectures per day covering basic endovascular intervention procedures but without the simulation training course.

Simulation system

The vascular simulator system (Angiomentor system, Simbionix, Ltd., Cleveland, OH, Fig 1) was composed of a standard desktop computer with software that simulated the human arterial system in 3 dimensions, and any user could perform the endovascular interventional procedures under the instruction of the system. This simulation system was connected to a haptic pressure feedback module, which used a force feedback system to detect external devices. When the users inserted the angiography catheters and wires, injected contrast and performed the endovascular procedures with digital subtraction angiography, all procedures could then be displayed on the screen in real time. The user was able to select the device to be simulated through one monitor, and the second monitor was used to display the simulated fluoroscopic image.

Procedure evaluation

For peer review only - http://bmjopen.bmj.com/site/about/guidelines.xhtml

When the residents completed the training course, all residents received assessments of theoretical knowledge at 1 month and practical assessments at 6 months. The teaching expert group made the theoretical test questions based on the key points of vascular disease as well as the endovascular interventional procedures and technical points involved in vascular surgery clinical training, and then the residents completed the assessment independently. Finally, the expert group determined the student's score based on the results of the test. The score range was 0-10, and a passing score was defined as a score higher than 7. All residents completed the arterial puncture procedure with the Seldinger technique and performed the cerebrovascular angiography procedure. The success criterion of arterial puncture was defined as follows: all puncture procedures were successfully completed, and the sheath was successfully inserted into the femoral artery. If the residents failed to complete the processes of puncture and sheath placement, which was defined as a puncture failure, then the puncture was performed by the teacher. The puncture success rate, puncture time and complications were recorded, and the comfort scores of the patients during the puncture procedure were assessed with a numerical rating scale (NRS). The NRS score ranged from 0 to 10, where 0 indicates worst uncomfortable pain and 10 indicates comfortable pain [7]. Subsequently, the residents needed to complete the cerebrovascular angiography procedure. The evaluation indicators of angiography were as following: learn time to complete procedure (LTP), time of complete procedure at the final test (TCP), fluo time of procedure (FTP), cumulative air kerma (CAK) and dose area product (DAP). LTP was defined as the time required from the beginning of training to the completion of the

For peer review only - http://bmjopen.bmj.com/site/about/guidelines.xhtml

first angiography procedure independently. TCP, FTP, CAK and DAP were defined as the time of procedure and related parameters of the assessed angiography procedure at the end of the training.

Patient and public involvement

Patients and the public were not involved in the design or planning of the study.

Statistical Analysis

All data were analyzed using SPSS v. 11.0 (SPSS, Chicago, IL, USA), and P <.05 was considered statistically significant. To test the difference between groups, we used Chi-square analysis for categorical variables and Student's t-tests for continuous variables, and we tested the significance of the difference between 2 independent proportions when the results were presented as percentages.

RESULTS

The baseline data of the two groups

This study included 48 residents and 47 residents who were retrospectively recruited in this study, and there was no significant difference in baseline data between the two groups (Table 1, P>0.05). There were 44 males and 4 females who received CT training, with a mean age of 33.13 years, and 42 males and 5 females who received ST training, with a mean age of 33.91 years (P>0.05). The background academic degrees were bachelor and postgraduate degrees in the CT and ST groups (P>0.05), and 30 and 26 residents had a specialty background in vascular surgery in both groups (62.50% vs 55.32%, P>0.05). The clinical work experience of most residents was less than three years in both groups (66.67% vs 57.45, P>0.05).

For peer review only - http://bmjopen.bmj.com/site/about/guidelines.xhtml

The theoretical scores between both groups

All residents passed the training test; however, the mean scores of the residents were higher in the ST group than in the CT group (8.74±1.09 vs 8.13±1.31, *P*=0.014). After the clinical training, the training satisfaction rates of all residents were similar (97.87% vs 91.67%, *P*>0.05); however, when asked whether they wished to participate in the training again, the residents in the ST group showed a higher willingness rate than residents in the CT groups (93.62% vs 79.17%, *P*=0.04).

The performance of arterial puncture in residents

After the training, all residents underwent an arterial puncture test (Table 2), and the success rate of the procedure was higher in the ST group than in the CT group (78.72% vs 58.33%, *P*=0.033); however, the total puncture success rate was similar between the two groups (95.74% vs 91.67%, *P*>0.05). The complications of the puncture sites included bruising, hematoma, infection and pseudoaneurysm, and there was no significant difference in the incidence of complications between the ST and CT groups (17.02% vs 18.75%, *P*>0.05); however, the time of the puncture procedure was shorter in the ST group than in the CT group (9.56±5.24 min vs 12.15±6.87 min, *P*=0.002), and the comfort score of patients was higher in the ST group than in the CT group according to the NRS scores (5.49±1.72 vs 4.71±1.57, *P*=0.023).

The outcome of cerebrovascular angiography

All residents needed to complete the final cerebrovascular angiography test; the related parameters are listed in Table 3. The residents in the ST group showed a shorter study curve with a lower mean LTP than residents in the CT groups (3.65 mon vs 4.07 mon,

For peer review only - http://bmjopen.bmj.com/site/about/guidelines.xhtml

$P<0.01$), and the TCP of the final test was higher in the CT group than in the ST group (41.32 min vs 33.81 min, $P=0.002$). The radiation damage-related parameters were recorded, and the residents in the CT group showed higher mean values of FTP (631.47 s vs 489.33 s, $P<0.001$) and CAK than the residents in the ST group (463.16 vs 401.30 mGy, $P=0.043$); however, the mean DAP value in both groups indicated no difference (128624.30 mGy.cm^2 vs 128012.10 mGy.cm^2, $P>0.052$).

DISCUSSION

The development of vascular surgery occurred relatively late in China; thus, the training model used for vascular residents in most university hospitals has been the CT model; however, traditional training did not improve residents' understanding and interest in vascular surgery. Therefore, in past decades, there were fewer residents who chose vascular surgery as a career option in China, which was consistent with the reports of previous studies [7]. Therefore, improving residents' interest in vascular surgery and promoting vascular clinical skills have been the main problems associated with vascular surgery training [8]. Earlier studies have shown that compared with traditional training, the use of network media, social media and simulation systems can achieve better training results [9-11]. In this study, we used the vascular simulation system to assist residents in clinical training. The results revealed that simulation training could significantly improve the clinical practice effect of residents. The residents who received the simulation training had significantly higher theoretical scores; in addition, the interest level of residents was higher after simulation training. The vascular simulation system could standardize complex vascular systems and different vascular

For peer review only - http://bmjopen.bmj.com/site/about/guidelines.xhtml

lesions through analog calculations, which could help residents practice vascular skills training earlier and improve the interest level and skills of residents [3].

After simulation training and basic training, residents need further vascular skills training. The basic skill procedure for vascular residents is arterial puncture. However, in clinical practice, it is impossible for residents to repeatedly perform the procedure during the treatment of patients. Therefore, in vitro simulation training has become the main teaching method in the training of vascular residents. It has been reported that simulation training can promote the clinical performance and vascular skills of residents [12]. Residents who received simulator training showed better clinical performance in the vascular surgery rotation, more motivation to learn, a shorter learning time and a lower number of clinical procedural errors, and the patients indicated a lower discomfort rate with the procedure [13]. In our study, the results confirmed that the residents who underwent the simulation training had a significantly higher success rate of arterial puncture, and the time of the puncture procedure was also significantly lower. Each step of the vascular procedure could be programmed and standardized in the simulation system. After the teacher's explanation and auxiliary training, it was easier for residents to develop standardized vascular skill habits and the correct procedural process. Finally, we evaluated the training effect with the cerebrovascular angiography procedure. Our results proved that the learning time of the angiography procedure and time of completed procedures were significantly lower in residents with simulation training; these results were consistent with previous reports [14]. This finding also confirmed that different simulation systems and teaching models could improve the

For peer review only - http://bmjopen.bmj.com/site/about/guidelines.xhtml

effectiveness of clinical teaching and promote the understanding and proficiency of vascular practical skills.

Furthermore, the final effect of clinical training should be assessed by the practice procedure of residents. Our study confirmed that vascular simulation training could significantly promote the practice skills of residents and promote the understanding of basic knowledge. Most cases of vascular disease teaching need to be performed under radiation; thus, simulation training could avoid radiation damage to teachers and residents. In this study, the results demonstrated that simulation training could decrease the fluo time and cumulative air kerma of the procedure, which meant that simulation training could reduce the radiation damage of patients and residents, and radiation protection was an important teaching ethics component in vascular surgery practice. However, due to the limitations of our teaching funding and the time course, only selective residents underwent the simulation training for 2 weeks, which was different from what occurs in advanced vascular centers. Other reports have shown that simulation training for 8 weeks can improve the procedure skills of residents, contribute to patient safety and have a positive impact on the career planning and choice of vascular surgeons [15].

In conclusion, our results confirmed that a vascular simulation system could improve the clinical skills of residents and reduce the radiation damage received by patients and residents in endovascular procedures. Nevertheless, this study was not a prospective randomized study, and simulation training was not used for every resident; thus, the conclusions of this study should be confirmed in the future.

For peer review only - http://bmjopen.bmj.com/site/about/guidelines.xhtml

Acknowledgments: This project was supported by the Natural Science Foundation of China (NO.8197 0365), and the language of this article was edited by American Journal Experts (AJE).

Conflicts of Interest: None.

Author Contributions: All authors made contributions to the conception and design of the paper. LY and YZ L contributed to the drafting of the work; LY, JLL and YML contributed to the critical revision of the paper. All authors approved the final manuscript for submission and have agreed to be accountable for all aspects of the paper.

Ethical approval: The study received institutional review board approval.

For peer review only

For peer review only - http://bmjopen.bmj.com/site/about/guidelines.xhtml

References:

[1] Song P, Rudan D, Wang M, Chang X, Rudan I. National and subnational estimation of the prevalence of peripheral artery disease (PAD) in China: a systematic review and meta-analysis. J Glob Health. 2019;9010601.

[2] Mills J. Vascular surgery training in the United States: A half-century of evolution. J Vasc Surg. 2008;48:90S-97S.

[3] Lee JT, Qiu M, Teshome M. The utility of endovascular simulation to improve technical performance and stimulate continued interest of preclinical medical students in vascular surgery. J Surg Educ, 2010, 66: 367-369.

[4] Aggarwal R, Black S, Hance J, et al. Virtual reality simulation training can improve inexperienced surgeons' endovascular skills. Eur J Vasc Endovasc Surg. 2007;31:588-593.

[5] Chaer RA, Derubertis BG, Lin SC, et al. Simulation improves resident performance in catheter-based intervention: Results of a randomized, controlled study. Ann Surg. 2006;244:343-352.

[6] Tedesco MM, Pak JJ, Harris EJ, et al. Simulation-based endovascular skills assessment: The future of credentialing? J Vasc Surg. 2008;47:1008-1001.

[7] Farrar JT, Young JP Jr, LaMoreaux L, Werth JL, Poole RM. Clinical importance of changes in chronic pain intensity measured on an 11-point numerical pain rating scale. Pain 2001; 94:149–158.

[8] Zimmerman P, O'Brien-Irr M, Dryjski M, et al. The new training paradigms and the

For peer review only - http://bmjopen.bmj.com/site/about/guidelines.xhtml

unfilled match positions of 2004: Will history repeat itself? J Vasc Surg. 2006; 44:145-150.

[9] Seymour N, Gallager A, Roman S, et al. Virtual reality training improves operating room performance: Results of a randomized, double-blind study. Ann Surg. 2002;236:458-463.

[10] Raval MV, Wang X, Cohen ME, et al. The influence of resident involvement on surgical outcomes. J Am Coll Surg. 2011;212:889-898.

[11] Smith BK, Greenberg JA, Mitchell EL. The evolving integrated vascular surgery residency curriculum. Ann Vasc Surg. 2014;28:1761-1768.

[12] Van Sickle K, Ritter E, Baghai M, et al. Prospective, randomized, double-blind trial of curriculum-based training for intracorporeal suturing and knot-tying. J Am Coll Surg. 2008;207: 560-568.

[13] Tan SS, Sarker SK. Simulation in surgery: a review. Scott Med J. 2011;56:104-109.

[14] Sturm LP, Windsor JA, Cosman PH, Cregan P, Hewett PJ, Maddern GJ. A systematic review of skills transfer after surgical simulation training. Ann Surg. 2008;248:166-179.

[15] Egle JP, Subramanyam Malladi SV, Gopinath N, Mittal VK. Simulation Training Improves Resident Performance in Hand-Sewn Vascular and Bowel Anastomoses. J Surg. 2014;72:291-296.

[16] Zayed MA, Lilo EA, Lee JT. Impact of an Interactive Vascular Surgery Web-Based Educational Curriculum on Surgical Trainee Knowledge and Interest. J

For peer review only

For peer review only - http://bmjopen.bmj.com/site/about/guidelines.xhtml

Surg Educ. 2016; 74:251-257.

Figure legends

Figure 1. The vascular simulator system (Angiomentor system, Simbionix, Ltd., Cleveland, OH) used in this study simulated the vascular interventional procedures of the cardiovascular, peripheral and cerebrovascular systems in 3 dimensions.

For peer review only

For peer review only - http://bmjopen.bmj.com/site/about/guidelines.xhtml

Tables

Table 1 The baseline data of both groups

	CT group (n=48)	ST group (n=47)	*P* value
Sex (M)	44 (91.67)	42 (89.36)	0.70
Age (years)	33.13±3.04	33.91±4.94	0.35
Academic degree			0.36
bachelor	22 (45.83)	26 (55.32)	
postgraduate	26 (54.17)	21 (44.68)	
Specialty background			0.48
vascular	30 (62.50)	26 (55.32)	
nonvascular	18 (37.50)	21 (44.68)	
Work experience			0.35
> 3 years	16 (33.33)	20 (42.55)	
≤ 3 years	32 (66.67)	27 (57.45)	

CT: conventional training; ST: simulation training; M: male.

P value, compared with the CT group

For peer review only - http://bmjopen.bmj.com/site/about/guidelines.xhtml

Table 2 The performance of arterial puncture in residents

	CT group (n=48)	ST group (n=47)	*P* value
Puncture success rate	28 (58.33)	37 (78.72)	0.033
Total puncture success rate	44 (91.67)	45 (95.74)	0.69
Complications from puncture	9 (18.75)	8 (17.02)	0.83
bruising	5 (10.42)	6 (12.77)	
hematoma	3 (6.25)	2 (4.26)	
infection	0	0	
arteriovenous fistula	0	0	
pseudoaneurysm	1 (2.08)	0	
time of puncture (min)	12.15±6.87	9.56±5.24	0.002
Comfort score of patients	4.71±1.57	5.49±1.72	0.023

CT: conventional training; ST: simulation training.

P value, compared with the CT group

For peer review only

Table 3 The performance on cerebrovascular angiography in residents

	CT group (n=48)	ST group (n=47)	*P* value
LTP (mon)	4.07±0.77	3.65±0.64	<0.01
TCP (min)	41.32±12.56	33.81±10.11	0.002
FTP (s)	631.47±243.65	489.33±237.13	0.005
CAK (mGy)	463.16±134.14	401.30±149.06	0.043
DAP (mGy.cm^2)	128624.30±28982.22	128012.10±31035.08	0.92

CT: conventional training; ST: simulation training; LTP: learn time to complete procedure from beginning; TCP: time of complete procedure at the final test; FTP: fluo time of procedure; CAK: cumulative air kerma; DAP: dose area product

P value, compared with the CT group

For peer review only

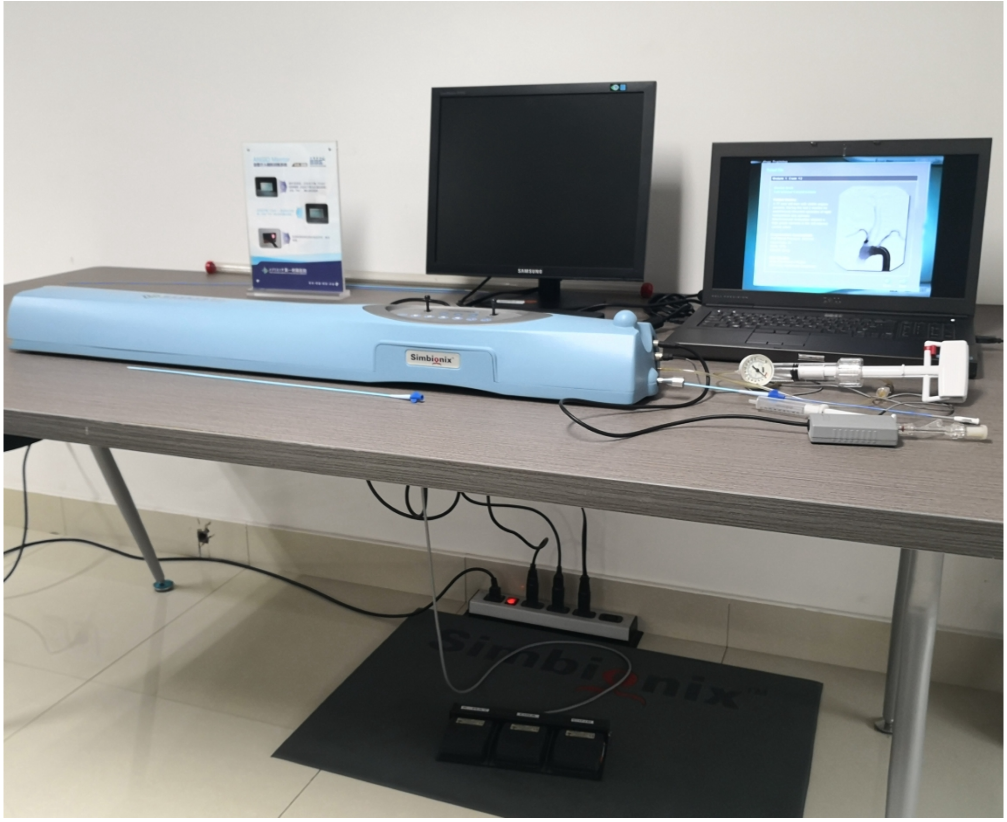

Figure 1. The vascular simulator system (Angiomentor system, Simbionix, Ltd., Cleveland, OH) used in this study simulated the vascular interventional procedures of the cardiovascular, peripheral and cerebrovascular systems in 3 dimensions.

1099x897mm (72 x 72 DPI)

STROBE 2007 (v4) checklist of items to be included in reports of observational studies in epidemiology*
Checklist for cohort, case-control, and cross-sectional studies (combined)

Section/Topic	Item #	Recommendation	Reported on page #
**Title and abstract**	1	(*a*) Indicate the study's design with a commonly used term in the title or the abstract	1–2
		(*b*) Provide in the abstract an informative and balanced summary of what was done and what was found	1–2
**Introduction**			
Background/rationale	2	Explain the scientific background and rationale for the investigation being reported	3–4
Objectives	3	State specific objectives, including any pre-specified hypotheses	3–4
**Methods**			
Study design	4	Present key elements of study design early in the paper	4–6
Setting	5	Describe the setting, locations, and relevant dates, including periods of recruitment, exposure, follow-up, and data collection	4–6
Participants	6	(*a*) *Cohort study*—Give the eligibility criteria, and the sources and methods of selection of participants. Describe methods of follow-up<br>*Case-control study*—Give the eligibility criteria, and the sources and methods of case ascertainment and control selection. Give the rationale for the choice of cases and controls<br>*Cross-sectional study*—Give the eligibility criteria, and the sources and methods of selection of participants	4–6
		(*b*) *Cohort study*—For matched studies, give matching criteria and number of exposed and unexposed<br>*Case-control study*—For matched studies, give matching criteria and the number of controls per case	4–6
Variables	7	Clearly define all outcomes, exposures, predictors, potential confounders, and effect modifiers. Give diagnostic criteria, if applicable	4–6
Data sources/ measurement	8*	For each variable of interest, give sources of data and details of methods of assessment (measurement). Describe comparability of assessment methods if there is more than one group	4–6
Bias	9	Describe any efforts to address potential sources of bias	4–6
Study size	10	Explain how the study size was arrived at	4–6
Quantitative variables	11	Explain how quantitative variables were handled in the analyses. If applicable, describe which groupings were chosen and why	4–6
Statistical methods	12	(*a*) Describe all statistical methods, including those used to control for confounding	7
		(*b*) Describe any methods used to examine subgroups and interactions	7
		(*c*) Explain how missing data were addressed	7
		(*d*) *Cohort study*—If applicable, explain how loss to follow-up was addressed<br>*Case-control study*—If applicable, explain how matching of cases and controls was addressed	7

For peer review only

		Cross-sectional study—If applicable, describe analytical methods taking account of sampling strategy	
		(*e*) Describe any sensitivity analyses	7
**Results**			
Participants	13*	(a) Report numbers of individuals at each stage of study—eg numbers potentially eligible, examined for eligibility, confirmed eligible, included in the study, completing follow-up, and analysed	7-9
		(b) Give reasons for non-participation at each stage	7-9
		(c) Consider use of a flow diagram	7-9
Descriptive data	14*	(a) Give characteristics of study participants (eg demographic, clinical, social) and information on exposures and potential confounders	7-9
		(b) Indicate number of participants with missing data for each variable of interest	7-9
		(c) *Cohort study*—Summarise follow-up time (eg, average and total amount)	7-9
Outcome data	15*	*Cohort study*—Report numbers of outcome events or summary measures over time	7-9
		Case-control study—Report numbers in each exposure category, or summary measures of exposure	7-9
		Cross-sectional study—Report numbers of outcome events or summary measures	7-9
Main results	16	(*a*) Give unadjusted estimates and, if applicable, confounder-adjusted estimates and their precision (eg, 95% confidence interval). Make clear which confounders were adjusted for and why they were included	7-9
		(*b*) Report category boundaries when continuous variables were categorized	7-9
		(*c*) If relevant, consider translating estimates of relative risk into absolute risk for a meaningful time period	7-9
Other analyses	17	Report other analyses done—eg analyses of subgroups and interactions, and sensitivity analyses	7-9
**Discussion**			
Key results	18	Summarise key results with reference to study objectives	9-11
Limitations	19	Discuss limitations of the study, taking into account sources of potential bias or imprecision. Discuss both direction and magnitude of any potential bias	9-11
Interpretation	20	Give a cautious overall interpretation of results considering objectives, limitations, multiplicity of analyses, results from similar studies, and other relevant evidence	9-11
Generalisability	21	Discuss the generalisability (external validity) of the study results	9-11
**Other information**			
Funding	22	Give the source of funding and the role of the funders for the present study and, if applicable, for the original study on which the present article is based	11

*Give information separately for cases and controls in case-control studies and, if applicable, for exposed and unexposed groups in cohort and cross-sectional studies.
Note: An Explanation and Elaboration article discusses each checklist item and gives methodological background and published examples of transparent reporting. The STROBE checklist is best used in conjunction with this article (freely available on the Web sites of PLoS Medicine at http://www.plosmedicine.org/, Annals of Internal Medicine at http://www.annals.org/, and Epidemiology at http://www.epidem.com/). Information on the STROBE Initiative is available at www.strobe-statement.org.

For peer review only

BMJ Open

The effect of vascular simulation training on practice performance in residents: a retrospective cohort study

Journal:	*BMJ Open*
Manuscript ID	bmjopen-2020-037338.R1
Article Type:	Original research
Date Submitted by the Author:	18-May-2020
Complete List of Authors:	Yang, Lin; First hoapital of Xi'an Jiaotong University, vascular surgery<br>Li, Yanzi; First hoapital of Xi'an Jiaotong University, Department of Medical Administration<br>Liu, Jianlin; First hoapital of Xi'an Jiaotong University, vascular surgery<br>Liu, Yamin; First hoapital of Xi'an Jiaotong University, international radiology
**Primary Subject Heading**:	Medical education and training
Secondary Subject Heading:	Medical education and training, Surgery
Keywords:	Vascular surgery < SURGERY, MEDICAL EDUCATION & TRAINING, VASCULAR MEDICINE

SCHOLARONE™
Manuscripts

I, the Submitting Author has the right to grant and does grant on behalf of all authors of the Work (as defined in the below author licence), an exclusive licence and/or a non-exclusive licence for contributions from authors who are: i) UK Crown employees; ii) where BMJ has agreed a CC-BY licence shall apply, and/or iii) in accordance with the terms applicable for US Federal Government officers or employees acting as part of their official duties; on a worldwide, perpetual, irrevocable, royalty-free basis to BMJ Publishing Group Ltd ("BMJ") its licensees and where the relevant Journal is co-owned by BMJ to the co-owners of the Journal, to publish the Work in this journal and any other BMJ products and to exploit all rights, as set out in our licence.

The Submitting Author accepts and understands that any supply made under these terms is made by BMJ to the Submitting Author unless you are acting as an employee on behalf of your employer or a postgraduate student of an affiliated institution which is paying any applicable article publishing charge ("APC") for Open Access articles. Where the Submitting Author wishes to make the Work available on an Open Access basis (and intends to pay the relevant APC), the terms of reuse of such Open Access shall be governed by a Creative Commons licence – details of these licences and which Creative Commons licence will apply to this Work are set out in our licence referred to above.

Other than as permitted in any relevant BMJ Author's Self Archiving Policies, I confirm this Work has not been accepted for publication elsewhere, is not being considered for publication elsewhere and does not duplicate material already published. I confirm all authors consent to publication of this Work and authorise the granting of this licence.

For peer review only

The effect of vascular simulation training on practice performance in residents: a retrospective cohort study

Lin Yang[1**], MD., Ph.D. Yanzi Li[2*], MD. Jianlin Liu[1], MD. Yamin Liu[3], MD

1. Department of Vascular Surgery, First Affiliated Hospital of Xi'an Jiaotong University, Xi'an, China
2. Department of Medical Administration, First Affiliated Hospital of Xi'an Jiaotong University, Xi'an, China
3. Department of Interventional Radiology, First Affiliated Hospital of Xi'an Jiaotong University, Xi'an, China

* Yanzi Li is the co-first author.

****Corresponding author:** LIN Yang. Department of Vascular Surgery, First Affiliated Hospital of Xi'an Jiaotong University, Xi'an, China. Tel.: +86 29 85324050. Fax: +86 29 85324050. *Email:* jdvascs@163.com

Running head: Effect of ST in residents

For peer review only - http://bmjopen.bmj.com/site/about/guidelines.xhtml

Abstract

Objective: This study aims to investigate the teaching effect of vascular simulation training in rotating vascular residents.

Design: Retrospective cohort study

Setting and participants: A total of 95 vascular residents were recruited from 2015 to 2018 in a university affiliated center western China, and divided into a simulation training (ST) group and a conventional training (CT) group. ST group received simulation training and conventional training, and the CT group only received conventional training.

Primary outcome measures: Theoretical scores were assessed, and the technique parameters, complications and radiation damage of the procedures were analyzed.

Results: The mean scores (8.74±1.09 vs 8.13±1.31) and the rate of willingness for retraining (93.62% vs 79.17%) in residents were higher in the ST group than in the CT group (P<0.05). The success rate of arterial puncture was significantly higher in the ST group (78.72% vs 58.33%, P=0.03); however, the incidence of complications was similar between the two groups (P>0.05). The time of the puncture procedure was significantly lower (9.56± 5.24 min vs 12.15±6.87 min), and the comfort score of the patient (5.49±1.72 vs 4.71±1.57) was higher in the ST group than in the CT group (P<0.05). At the end of the assessment, the learning time for angiography (3.65±0.64 mon vs 4.07±0.77 mon) and the complete procedure time (33.81±10.11 min vs 41.32±12.56 min) were lower in the ST group than in the CT group (P<0.01). The fluo time for angiography (489.33±237.13s vs 631.47±243.65 s) and the cumulative air

For peer review only

For peer review only - http://bmjopen.bmj.com/site/about/guidelines.xhtml

kerma (401.30±149.06 mGy vs 461.16±134.14 mGy) were significantly decreased in ST group (*P*<0.05).

Conclusion: The application of a vascular simulation system can significantly improve the clinical performance of residents and reduce the radiation damage from a single intervention procedure in patients.

Keywords: Vascular surgery; Medical education & training; Teaching modes; Simulation training; Traditional teaching

For peer review only

For peer review only - http://bmjopen.bmj.com/site/about/guidelines.xhtml

Article summary

Strengths and limitations of this study

- The simulation training could promote the mean scores of residents
- The simulation training could improve the clinical performance of residents
- The simulation training could reduce the radiation damage
- The simulation training should be wildly used in clinical teaching practice.
- The conclusions of this study should be confirmed via prospective randomized study.

For peer review only

For peer review only - http://bmjopen.bmj.com/site/about/guidelines.xhtml

INTRODUCTION

In recent years, with changes in the disease spectrum of Chinese patients, the incidence of peripheral arterial disease has increased significantly, which has also caused a severe economic and social burden [1]. Therefore, it is becoming increasingly important to strengthen general and specialized vascular disease skills education in the training of medical residents [2, 3]. In the past decade, the practical skills training of resident has been mainly carried out through conventional teaching (CT) modes (including classroom teaching, lectures and surgical practice), and residents lack sufficient practical procedures with simulation training from theoretical knowledge to practical procedures, therefore, the true teaching effect was not ideal.

Three-dimensional vascular simulator systems (Angiomentor system, Simbionix, Ltd., Cleveland, OH) use digital simulation to quantify the vascular interventional procedures of the cardiovascular, peripheral and cerebrovascular systems. Residents can use the system to select cases for simulation training; ultimately, the simulation training results are scored according to the operating steps of the system. This simulation training can promote the mastery of vascular procedure performance in residents [4-6], and this system provides an opportunity to perform endovascular procedures in a safe environment as an educational tool for novice residents.

However, due to the late entry of simulation systems in China, there has been no report of the use of 3-dimensional vascular simulation systems in clinical practice teaching in the area of vascular surgery. The aim of this study was to assess the effect of simulation-based training on improving the technical performance and subsequent

For peer review only - http://bmjopen.bmj.com/site/about/guidelines.xhtml

clinical procedures of residents in vascular surgery.

METHODS

Study procedures

A total of 95 vascular resident trainees at the First Affiliated Hospital of Xi'an Jiaotong University were respectively collected in this study from Jan 2015 to Dec 2018, 47 vascular residents received simulation-based vascular training (ST group) for two weeks, and then they completed the last clinical training. The other 48 residents only completed conventional clinical training (CT group, including classroom teaching, lectures and surgical practice) without the simulation course, and all residents needed to complete 6 months of endovascular training in vascular surgery. A survey was administered to determine the demographics, academic degree, specialty level, and previous work experiences that may have been relevant in terms of the residents' ability to learn vascular interventional skills. This study was approved by the institutional review and ethics board of the First Affiliated Hospital of Xi'an Jiaotong University (XJTU1AF2014LSK-112), all of the patients provided written informed consent.

Before the course was performed, the residents in the ST group received a standardized introduction to the endovascular simulator and performed a cerebrovascular angiography procedure. The 2-week curriculum consisted of theory teaching and 30-60 min lectures per day covering basic catheter-based interventions, cerebrovascular disease, superficial femoral artery disease and renal artery disease. The residents received 1-hour mentored simulator sessions per day and practiced carotid, renal and superficial femoral artery interventions. This course was performed by a tutor.

For peer review only - http://bmjopen.bmj.com/site/about/guidelines.xhtml

Then, residents needed to complete the primary endovascular procedure with direct instruction. During the entire training process, each resident was required to complete the simulation training for no less than 1 hour per day. The course concluded with a final cerebrovascular angiography procedure performed on the simulator. The residents in the CT group underwent the basic 2-week curriculum consisting of theory teaching and 30-60 min lectures per day covering basic endovascular intervention procedures but without the simulation training course.

Simulation system

The vascular simulator system (Angiomentor system, Simbionix, Ltd., Cleveland, OH, Fig 1) was composed of a standard desktop computer with software that simulated the human arterial system in 3 dimensions, and any user could perform the endovascular interventional procedures under the instruction of the system. This simulation system was connected to a haptic pressure feedback module, which used a force feedback system to detect external devices. When the users inserted the angiography catheters and wires, injected contrast and performed the endovascular procedures with digital subtraction angiography, all procedures could then be displayed on the screen in real time. The user was able to select the device to be simulated through one monitor, and the second monitor was used to display the simulated fluoroscopic image.

Procedure evaluation

When the residents completed the training course, all residents received assessments of theoretical knowledge at 1 month and practical assessments at 6 months. The teaching expert group made the theoretical test questions based on the key points of vascular

For peer review only - http://bmjopen.bmj.com/site/about/guidelines.xhtml

disease as well as the endovascular interventional procedures and technical points involved in vascular surgery clinical training, and then the residents completed the assessment independently. Finally, the expert group determined the student's score based on the results of the test (including the detailed information of knowledge and practice). The theoretical score range was 0-10, and a passing score was defined as a score higher than 7. All residents completed the arterial puncture procedure with the Seldinger technique and performed the cerebrovascular angiography procedure. The success criterion of arterial puncture was defined as follows: all puncture procedures were successfully completed, and the sheath was successfully inserted into the femoral artery. If the residents failed to complete the processes of puncture and sheath placement, which was defined as a puncture failure, then the puncture was performed by the teacher. The puncture success rate, puncture time and complications were recorded, and the comfort scores of the patients during the puncture procedure were assessed with a numerical rating scale (NRS). The NRS score ranged from 0 to 10, where 0 indicates worst uncomfortable pain and 10 indicates comfortable pain [7]. Subsequently, the residents needed to complete the cerebrovascular angiography procedure. The evaluation indicators of angiography were as following: learn time to complete the procedure (LTP, defined as the time from the beginning of training to the completion of the first angiography independently), time of complete procedure at the final test (TCP), fluo time of procedure (FTP), cumulative air kerma (CAK) and dose area product (DAP). TCP, FTP, CAK and DAP were defined as the time of procedure and related parameters of the assessed angiography procedure at the end of the training.

For peer review only - http://bmjopen.bmj.com/site/about/guidelines.xhtml

Patient and public involvement

Patients and the public were not involved in the design or planning of the study.

Statistical Analysis

All data were analyzed using SPSS v. 11.0 (SPSS, Chicago, IL, USA), and $P < .05$ was considered statistically significant. To test the difference between groups, we used Chi-square analysis for categorical variables and Student's t-tests for continuous variables, and we tested the significance of the difference between 2 independent proportions when the results were presented as percentages.

RESULTS

The baseline data of the two groups

This study included 48 residents and 47 residents who were retrospectively recruited in this study, and there was no significant difference in baseline data between the two groups (Table 1, $P>0.05$). There were 44 males and 4 females who received CT training, with a mean age of 33.13 years, and 42 males and 5 females who received ST training, with a mean age of 33.91 years ($P>0.05$). The background academic degrees were bachelor and postgraduate degrees in the CT and ST groups ($P>0.05$), and 30 and 26 residents had a specialty background in vascular surgery in both groups (62.50% vs 55.32%, $P>0.05$). The clinical work experience of most residents was less than three years in both groups (66.67% vs 57.45, $P>0.05$).

The theoretical scores between both groups

All residents passed the training test; however, the mean scores of the residents were higher in the ST group than in the CT group (8.74±1.09 vs 8.13±1.31, $P=0.014$). After

For peer review only - http://bmjopen.bmj.com/site/about/guidelines.xhtml

the clinical training, the training satisfaction rates of all residents were similar (97.87% vs 91.67%, $P>0.05$); however, when asked whether they wished to participate in the training again, the residents in the ST group showed a higher willingness rate than residents in the CT groups (93.62% vs 79.17%, $P=0.04$).

The performance of arterial puncture in residents

After the training, all residents underwent an arterial puncture test (Table 2), and the success rate of the procedure was higher in the ST group than in the CT group (78.72% vs 58.33%, $P=0.033$); however, the total puncture success rate was similar between the two groups (95.74% vs 91.67%, $P>0.05$). The complications of the puncture sites included bruising, hematoma, infection and pseudoaneurysm, and there was no significant difference in the incidence of complications between the ST and CT groups (17.02% vs 18.75%, $P>0.05$); however, the time of the puncture procedure was shorter in the ST group than in the CT group (9.56±5.24 min vs 12.15±6.87 min, $P=0.002$), and the comfort score of patients was higher in the ST group than in the CT group according to the NRS scores (5.49±1.72 vs 4.71±1.57, $P=0.023$).

The outcome of cerebrovascular angiography

All residents needed to complete the final cerebrovascular angiography test; the related parameters are listed in Table 3. The residents in the ST group showed a shorter study curve with a lower mean LTP than residents in the CT groups (3.65 mon vs 4.07 mon, $P<0.01$), and the TCP of the final test was higher in the CT group than in the ST group (41.32 min vs 33.81 min, $P=0.002$). The radiation damage-related parameters were recorded, and the residents in the CT group showed higher mean values of FTP (631.47

For peer review only - http://bmjopen.bmj.com/site/about/guidelines.xhtml

s vs 489.33 s, *P*<0.001) and CAK than the residents in the ST group (463.16 vs 401.30 mGy, *P*=0.043); however, the mean DAP value in both groups indicated no difference (128624.30 mGy.cm^2 vs 128012.10 mGy.cm^2, *P*>0.052).

DISCUSSION

The development of vascular surgery occurred relatively late in China; thus, the training model used for vascular residents in most university hospitals was traditional training; however, traditional training did not improve residents' understanding and interest in vascular surgery. In past decades, there were fewer residents who chose vascular surgery as a career option in China, which was consistent with the reports of previous studies [7]. Therefore, improving residents' interest in vascular surgery and promoting vascular clinical performance have been the main problems associated with clinical training [8]. Earlier studies have shown that compared with traditional training, the use of network media, social media and simulation systems can achieve better training results [9-11]. In this study, we used the vascular simulation system to assist residents in clinical training. The results revealed that simulation training could significantly improve the clinical practice effect of residents. The residents who received the simulation training had significantly higher theoretical scores; in addition, the interest level of residents was higher after simulation training. The vascular simulation system could standardize complex vascular systems and different vascular lesions through analog calculations, which could help residents practice vascular skills training earlier and improve the interest and performance of residents [3]. Our result reveals that the residents could deeply understand theoretical knowledge and practical knowledge

For peer review only - http://bmjopen.bmj.com/site/about/guidelines.xhtml

through simulation training, which also helps to improve the residents' theoretical and practical knowledge.

After simulation training and basic training, residents need further vascular operation training under the guidance of tutors, and the basic procedure for vascular residents is arterial puncture. However, it is impossible for residents to repeatedly perform the procedure during the treatment of patients; thus, in vitro simulation training has become the main teaching method in the training of vascular residents. Residents who received simulator training showed better clinical performance in the vascular surgery rotation, more motivation to learn, a shorter learning time and a lower number of clinical procedural errors, and the patients indicated a lower discomfort rate with the procedure [12, 13]. In our study, the results confirmed that the residents who underwent the simulation training had a significantly higher success rate of arterial puncture, and the time of the puncture procedure was also significantly lower. Each step of the vascular procedure could be programmed and standardized in the simulation system. After the teacher's explanation and auxiliary training, it was easier for residents to develop standardized vascular skill habits and the correct procedural process. Finally, we evaluated the training effect with the cerebrovascular angiography procedure. Our results proved that the learning time of the angiography procedure and time of completed procedures were significantly lower in residents with simulation training; these results were consistent with previous reports [14]. This finding also confirmed that different simulation systems and teaching models could improve the effectiveness of clinical teaching and promote the understanding and proficiency of vascular practical

For peer review only - http://bmjopen.bmj.com/site/about/guidelines.xhtml

performance.

Furthermore, the final effect of clinical training should be assessed by the practice procedure of residents. Our study confirmed that vascular simulation training could promote the practice knowledge of residents and promote the understanding of vascular surgical procedure. Most cases of vascular disease teaching need to be performed under radiation; thus, simulation training could avoid radiation damage to teachers, patients and residents. In this study, the results demonstrated that simulation training could decrease the fluo time and cumulative air kerma of the procedure, which meant that simulation training could reduce the radiation damage of patients and residents, and radiation protection was an important teaching ethics component in vascular surgery practice. However, due to the limitations of our teaching funding and the time course, only selective residents underwent the simulation training for 2 weeks, which was different from what occurs in advanced vascular centers. Other reports have shown that simulation training for 8 weeks can improve the procedure skills of residents, contribute to patient safety and have a positive impact on the career planning and choice of vascular surgeons [15]. Therefore, the simulation training should be the basic course for residents. Therefore, the simulation training should be the basic course for residents, especially in pre-career students and residents in rotation, meanwhile the simulation training should be well-defined and step-planned, otherwise, the simulation training may result in an impaired learning and worse performance in real clinical practice [16].

Limitations

There were several limitations. First, this study was not a prospective randomized study,

For peer review only - http://bmjopen.bmj.com/site/about/guidelines.xhtml

For peer review only

and the conclusions of this study should be confirmed in the future; Second, when compared with Western counties, the simulation training is not wildly used in China, thus the truly effect of the simulation training is not investigated deeply; Third, the clinical performance of the residents should be evaluated via the real clinical procedure after the simulation training, this is the next step of our study.

Conclusions

Our results confirmed that a vascular simulation system could improve the clinical skills of residents and reduce the radiation damage received by patients and residents in endovascular procedures.

Author Contributions: LY, YZ L: data collection. LY, YZ L, JL L and YM L: conception or design of the work; data analysis and interpretation; critical revision of the article; drafting of the article and final approval of the version to be published.

Acknowledgments: The language of this article was edited by American Journal Experts (AJE).

Funding: This project was supported by the Natural Science Foundation of China (NO.8197 0365)

Conflicts of Interest: None.

Patient consent for publication: Not required.

Ethical approval: This study was approved by the institutional review and ethics board of the First Affiliated Hospital of Xi'an Jiaotong University.

Data availability statement: Data are available upon reasonable request.

For peer review only - http://bmjopen.bmj.com/site/about/guidelines.xhtml

References:

[1] Song P, Rudan D, Wang M, Chang X, Rudan I. National and subnational estimation of the prevalence of peripheral artery disease (PAD) in China: a systematic review and meta-analysis. J Glob Health. 2019;9010601.

[2] Mills J. Vascular surgery training in the United States: A half-century of evolution. J Vasc Surg. 2008;48:90S-97S.

[3] Lee JT, Qiu M, Teshome M. The utility of endovascular simulation to improve technical performance and stimulate continued interest of preclinical medical students in vascular surgery. J Surg Educ, 2010, 66: 367-369.

[4] Aggarwal R, Black S, Hance J, et al. Virtual reality simulation training can improve inexperienced surgeons' endovascular skills. Eur J Vasc Endovasc Surg. 2007;31:588-593.

[5] Chaer RA, Derubertis BG, Lin SC, et al. Simulation improves resident performance in catheter-based intervention: Results of a randomized, controlled study. Ann Surg. 2006;244:343-352.

[6] Tedesco MM, Pak JJ, Harris EJ, et al. Simulation-based endovascular skills assessment: The future of credentialing? J Vasc Surg. 2008;47:1008-1001.

[7] Farrar JT, Young JP Jr, LaMoreaux L, Werth JL, Poole RM. Clinical importance of changes in chronic pain intensity measured on an 11-point numerical pain rating scale. Pain 2001; 94:149–158.

[8] Zimmerman P, O'Brien-Irr M, Dryjski M, et al. The new training paradigms and the unfilled match positions of 2004: Will history repeat itself? J Vasc Surg. 2006;

For peer review only

For peer review only - http://bmjopen.bmj.com/site/about/guidelines.xhtml

44:145-150.

[9] Seymour N, Gallager A, Roman S, et al. Virtual reality training improves operating room performance: Results of a randomized, double-blind study. Ann Surg. 2002;236:458-463.

[10] Raval MV, Wang X, Cohen ME, et al. The influence of resident involvement on surgical outcomes. J Am Coll Surg. 2011;212:889-898.

[11] Smith BK, Greenberg JA, Mitchell EL. The evolving integrated vascular surgery residency curriculum. Ann Vasc Surg. 2014;28:1761-1768.

[12] Van Sickle K, Ritter E, Baghai M, et al. Prospective, randomized, double-blind trial of curriculum-based training for intracorporeal suturing and knot-tying. J Am Coll Surg. 2008;207: 560-568.

[13] Tan SS, Sarker SK. Simulation in surgery: a review. Scott Med J. 2011;56:104-109.

[14] Sturm LP, Windsor JA, Cosman PH, Cregan P, Hewett PJ, Maddern GJ. A systematic review of skills transfer after surgical simulation training. Ann Surg. 2008;248:166-179.

[15] Egle JP, Subramanyam Malladi SV, Gopinath N, Mittal VK. Simulation Training Improves Resident Performance in Hand-Sewn Vascular and Bowel Anastomoses. J Surg. 2014;72:291-296.

[16] Jensen UJ, Jensen J, Olivecrona G, Ahlberg G, Lagerquist B, Tornvall P. The role of a simulator-based course in coronary angiography on performance in real life cath lab. BMC Med Educ. 2014;14:49.

For peer review only

For peer review only - http://bmjopen.bmj.com/site/about/guidelines.xhtml

Figure legends

Figure 1. The vascular simulator system (Angiomentor system, Simbionix, Ltd., Cleveland, OH) used in this study simulated the vascular interventional procedures: a) work station; b) pedal; c) simulator.

For peer review only

For peer review only - http://bmjopen.bmj.com/site/about/guidelines.xhtml

Tables

Table 1 The baseline data of both groups

	CT group (n=48)	ST group (n=47)	*P* value
Sex (M)	44 (91.67)	42 (89.36)	0.70
Age (years)	33.13±3.04	33.91±4.94	0.35
Academic degree			0.36
bachelor	22 (45.83)	26 (55.32)	
postgraduate	26 (54.17)	21 (44.68)	
Specialty background			0.48
vascular	30 (62.50)	26 (55.32)	
nonvascular	18 (37.50)	21 (44.68)	
Work experience			0.35
> 3 years	16 (33.33)	20 (42.55)	
≤ 3 years	32 (66.67)	27 (57.45)	

CT: conventional training; ST: simulation training; M: male.

P value, compared with the CT group

For peer review only - http://bmjopen.bmj.com/site/about/guidelines.xhtml

Table 2 The performance of arterial puncture in residents

	CT group (n=48)	ST group (n=47)	*P* value
Puncture success rate	28 (58.33)	37 (78.72)	0.033
Total puncture success rate	44 (91.67)	45 (95.74)	0.69
Complications from puncture	9 (18.75)	8 (17.02)	0.83
bruising	5 (10.42)	6 (12.77)	
hematoma	3 (6.25)	2 (4.26)	
infection	0	0	
arteriovenous fistula	0	0	
pseudoaneurysm	1 (2.08)	0	
time of puncture (min)	12.15±6.87	9.56±5.24	0.002
Comfort score of patients	4.71±1.57	5.49±1.72	0.023

CT: conventional training; ST: simulation training.

P value, compared with the CT group

For peer review only

For peer review only - http://bmjopen.bmj.com/site/about/guidelines.xhtml

Table 3 The performance on cerebrovascular angiography in residents

	CT group (n=48)	ST group (n=47)	*P* value
LTP (mon)	4.07±0.77	3.65±0.64	<0.01
TCP (min)	41.32±12.56	33.81±10.11	0.002
FTP (s)	631.47±243.65	489.33±237.13	0.005
CAK (mGy)	463.16±134.14	401.30±149.06	0.043
DAP ($mGy.cm^2$)	128624.30±28982.22	128012.10±31035.08	0.92

CT: conventional training; ST: simulation training; LTP: learn time to complete procedure from beginning; TCP: time of complete procedure at the final test; FTP: fluo time of procedure; CAK: cumulative air kerma; DAP: dose area product

P value, compared with the CT group

For peer review only

For peer review only - http://bmjopen.bmj.com/site/about/guidelines.xhtml

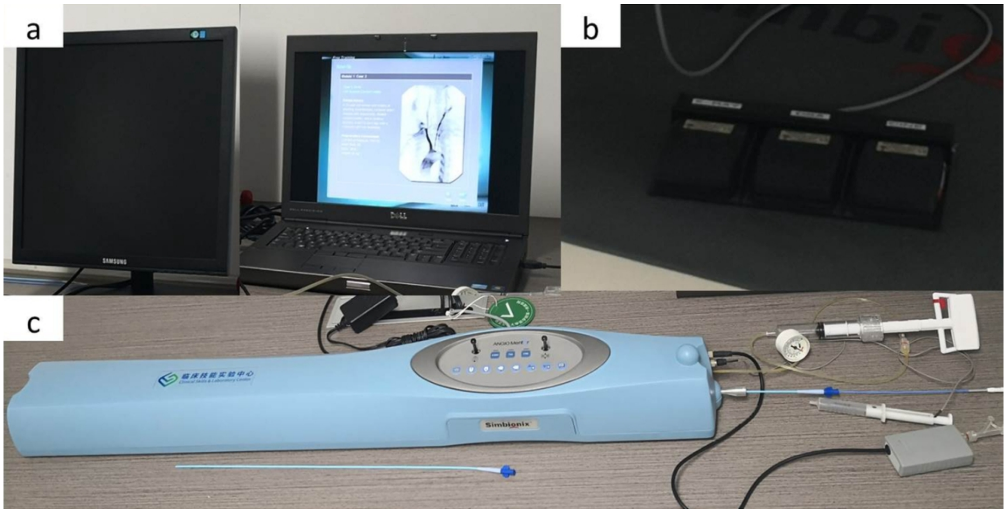

Figure 1. The vascular simulator system (Angiomentor system, Simbionix, Ltd., Cleveland, OH) used in this study simulated the vascular interventional procedures: a) work station; b) pedal; c) simulator.

124x63mm (300 x 300 DPI)

STROBE 2007 (v4) checklist of items to be included in reports of observational studies in epidemiology*
Checklist for cohort, case-control, and cross-sectional studies (combined)

Section/Topic	Item #	Recommendation	Reported on page #
**Title and abstract**	1	(*a*) Indicate the study's design with a commonly used term in the title or the abstract	1–2
		(*b*) Provide in the abstract an informative and balanced summary of what was done and what was found	1–2
**Introduction**			
Background/rationale	2	Explain the scientific background and rationale for the investigation being reported	3–4
Objectives	3	State specific objectives, including any pre-specified hypotheses	3–4
**Methods**			
Study design	4	Present key elements of study design early in the paper	4–6
Setting	5	Describe the setting, locations, and relevant dates, including periods of recruitment, exposure, follow-up, and data collection	4–6
Participants	6	(*a*) *Cohort study*—Give the eligibility criteria, and the sources and methods of selection of participants. Describe methods of follow-up<br>*Case-control study*—Give the eligibility criteria, and the sources and methods of case ascertainment and control selection. Give the rationale for the choice of cases and controls<br>*Cross-sectional study*—Give the eligibility criteria, and the sources and methods of selection of participants	4–6
		(*b*) *Cohort study*—For matched studies, give matching criteria and number of exposed and unexposed<br>*Case-control study*—For matched studies, give matching criteria and the number of controls per case	4–6
Variables	7	Clearly define all outcomes, exposures, predictors, potential confounders, and effect modifiers. Give diagnostic criteria, if applicable	4–6
Data sources/ measurement	8*	For each variable of interest, give sources of data and details of methods of assessment (measurement). Describe comparability of assessment methods if there is more than one group	4–6
Bias	9	Describe any efforts to address potential sources of bias	4–6
Study size	10	Explain how the study size was arrived at	4–6
Quantitative variables	11	Explain how quantitative variables were handled in the analyses. If applicable, describe which groupings were chosen and why	4–6
Statistical methods	12	(*a*) Describe all statistical methods, including those used to control for confounding	7
		(*b*) Describe any methods used to examine subgroups and interactions	7
		(*c*) Explain how missing data were addressed	7
		(*d*) *Cohort study*—If applicable, explain how loss to follow-up was addressed<br>*Case-control study*—If applicable, explain how matching of cases and controls was addressed	7

		Cross-sectional study—If applicable, describe analytical methods taking account of sampling strategy	
		(*e*) Describe any sensitivity analyses	7
**Results**			
Participants	13*	(a) Report numbers of individuals at each stage of study—eg numbers potentially eligible, examined for eligibility, confirmed eligible, included in the study, completing follow-up, and analysed	7–9
		(b) Give reasons for non-participation at each stage	7–9
		(c) Consider use of a flow diagram	7–9
Descriptive data	14*	(a) Give characteristics of study participants (eg demographic, clinical, social) and information on exposures and potential confounders	7–9
		(b) Indicate number of participants with missing data for each variable of interest	7–9
		(c) *Cohort study*—Summarise follow-up time (eg, average and total amount)	7–9
Outcome data	15*	*Cohort study*—Report numbers of outcome events or summary measures over time	7–9
		Case-control study—Report numbers in each exposure category, or summary measures of exposure	7–9
		Cross-sectional study—Report numbers of outcome events or summary measures	7–9
Main results	16	(*a*) Give unadjusted estimates and, if applicable, confounder-adjusted estimates and their precision (eg, 95% confidence interval). Make clear which confounders were adjusted for and why they were included	7–9
		(*b*) Report category boundaries when continuous variables were categorized	7–9
		(*c*) If relevant, consider translating estimates of relative risk into absolute risk for a meaningful time period	7–9
Other analyses	17	Report other analyses done—eg analyses of subgroups and interactions, and sensitivity analyses	7–9
**Discussion**			
Key results	18	Summarise key results with reference to study objectives	9–11
Limitations	19	Discuss limitations of the study, taking into account sources of potential bias or imprecision. Discuss both direction and magnitude of any potential bias	9–11
Interpretation	20	Give a cautious overall interpretation of results considering objectives, limitations, multiplicity of analyses, results from similar studies, and other relevant evidence	9–11
Generalisability	21	Discuss the generalisability (external validity) of the study results	9–11
**Other information**			
Funding	22	Give the source of funding and the role of the funders for the present study and, if applicable, for the original study on which the present article is based	11

*Give information separately for cases and controls in case-control studies and, if applicable, for exposed and unexposed groups in cohort and cross-sectional studies.

Note: An Explanation and Elaboration article discusses each checklist item and gives methodological background and published examples of transparent reporting. The STROBE checklist is best used in conjunction with this article (freely available on the Web sites of PLoS Medicine at http://www.plosmedicine.org/, Annals of Internal Medicine at http://www.annals.org/, and Epidemiology at http://www.epidem.com/). Information on the STROBE Initiative is available at www.strobe-statement.org.

For peer review only

BMJ Open

The effect of vascular simulation training on practice performance in residents: a retrospective cohort study

Journal:	*BMJ Open*
Manuscript ID	bmjopen-2020-037338.R2
Article Type:	Original research
Date Submitted by the Author:	27-Jul-2020

Complete List of Authors:	Yang, Lin; Xi'an Jiaotong University Medical College First Affiliated Hospital, vascular surgery<br>Li, Yanzi; Xi'an Jiaotong University Medical College First Affiliated Hospital, Department of Medical Administration<br>Liu, Jianlin; Xi'an Jiaotong University Medical College First Affiliated Hospital, vascular surgery<br>Liu, Yamin; Xi'an Jiaotong University Medical College First Affiliated Hospital, international radiology

**Primary Subject Heading**:	Medical education and training
Secondary Subject Heading:	Medical education and training, Surgery
Keywords:	Vascular surgery < SURGERY, MEDICAL EDUCATION & TRAINING, VASCULAR MEDICINE

SCHOLARONE™
Manuscripts

For peer review only - http://bmjopen.bmj.com/site/about/guidelines.xhtml

I, the Submitting Author has the right to grant and does grant on behalf of all authors of the Work (as defined in the below author licence), an exclusive licence and/or a non-exclusive licence for contributions from authors who are: i) UK Crown employees; ii) where BMJ has agreed a CC-BY licence shall apply, and/or iii) in accordance with the terms applicable for US Federal Government officers or employees acting as part of their official duties; on a worldwide, perpetual, irrevocable, royalty-free basis to BMJ Publishing Group Ltd ("BMJ") its licensees and where the relevant Journal is co-owned by BMJ to the co-owners of the Journal, to publish the Work in this journal and any other BMJ products and to exploit all rights, as set out in our licence.

The Submitting Author accepts and understands that any supply made under these terms is made by BMJ to the Submitting Author unless you are acting as an employee on behalf of your employer or a postgraduate student of an affiliated institution which is paying any applicable article publishing charge ("APC") for Open Access articles. Where the Submitting Author wishes to make the Work available on an Open Access basis (and intends to pay the relevant APC), the terms of reuse of such Open Access shall be governed by a Creative Commons licence – details of these licences and which Creative Commons licence will apply to this Work are set out in our licence referred to above.

Other than as permitted in any relevant BMJ Author's Self Archiving Policies, I confirm this Work has not been accepted for publication elsewhere, is not being considered for publication elsewhere and does not duplicate material already published. I confirm all authors consent to publication of this Work and authorise the granting of this licence.

For peer review only

The effect of vascular simulation training on practice performance in residents: a retrospective cohort study

Lin Yang[1**], MD., Ph.D. Yanzi Li[2*], MD. Jianlin Liu[1], MD. Yamin Liu[3], MD

1. Department of Vascular Surgery, First Affiliated Hospital of Xi'an Jiaotong University, Xi'an, China
2. Department of Medical Administration, First Affiliated Hospital of Xi'an Jiaotong University, Xi'an, China
3. Department of Interventional Radiology, First Affiliated Hospital of Xi'an Jiaotong University, Xi'an, China

* Yanzi Li is the co-first author.

****Corresponding author:** LIN Yang. Department of Vascular Surgery, First Affiliated Hospital of Xi'an Jiaotong University, Xi'an, China. Tel.: +86 29 85324050. Fax: +86 29 85324050. *Email:* jdvascs@163.com

Running head: Effect of ST in residents

For peer review only - http://bmjopen.bmj.com/site/about/guidelines.xhtml

Abstract

Objective: This study aims to investigate the teaching effect of vascular simulation training in rotating vascular residents.

Design: Retrospective cohort study

Setting and participants: A total of 95 vascular residents were included from 2015 to 2018 in a university affiliated center western China, and divided into a simulation training (ST) group and a conventional training (CT) group. ST group received simulation training and conventional training, and the CT group only received conventional training.

Primary outcome measures: Theoretical scores were assessed, and the technique parameters, complications and radiation damage of the procedures were analyzed.

Results: The mean scores (8.74±1.09 vs 8.13±1.31) and the rate of willingness for retraining (93.62% vs 79.17%) in residents were higher in the ST group than in the CT group (*P*<0.05). The success rate of arterial puncture was significantly higher in the ST group (78.72% vs 58.33%, *P*=0.03); however, the incidence of complications was similar between the two groups (*P*>0.05). The time of the puncture procedure was significantly lower (9.56± 5.24 min vs 12.15±6.87 min), and the comfort score of the patient (5.49±1.72 vs 4.71±1.57) was higher in the ST group than in the CT group (*P*<0.05). At the end of the assessment, the learning time for angiography (3.65±0.64 mon vs 4.07±0.77 mon) and the complete procedure time (33.81±10.11 min vs 41.32±12.56 min) were lower in the ST group than in the CT group (*P*<0.01). The fluo time for angiography (489.33±237.13s vs 631.47±243.65 s) and the cumulative air

For peer review only - http://bmjopen.bmj.com/site/about/guidelines.xhtml

kerma (401.30±149.06 mGy vs 461.16±134.14 mGy) were significantly decreased in ST group (P<0.05).

Conclusion: The application of a vascular simulation system can significantly improve the clinical performance of residents and reduce the radiation damage from a single intervention procedure in patients.

Keywords: Vascular surgery; Medical education & training; Teaching modes; Simulation training; Traditional teaching

For peer review only

Article summary

Strengths and limitations of this study

- The simulation training could promote the mean scores of residents
- The simulation training could improve the clinical performance of residents
- The simulation training could reduce the radiation damage
- The simulation training should be wildly used in clinical teaching practice.
- The conclusions of this study should be confirmed via prospective randomized study.

For peer review only - http://bmjopen.bmj.com/site/about/guidelines.xhtml

INTRODUCTION

In recent years, with changes in the disease spectrum of Chinese patients, the incidence of peripheral arterial disease has increased significantly, which has also caused a severe economic and social burden [1]. Therefore, it is becoming increasingly important to strengthen general and specialized vascular disease skills education in the training of medical residents [2, 3]. In the past decade, the practical skills training of resident has been mainly carried out through conventional teaching (CT) modes (including classroom teaching, lectures and surgical practice), and residents lack sufficient practical procedures with simulation training from theoretical knowledge to practical procedures, therefore, the true teaching effect was not ideal.

Three-dimensional vascular simulator systems (Angiomentor system, Simbionix, Ltd., Cleveland, OH) use digital simulation to quantify the vascular interventional procedures of the cardiovascular, peripheral and cerebrovascular systems. Residents can use the system to select cases for simulation training; ultimately, the simulation training results are scored according to the operating steps of the system. This simulation training can promote the mastery of vascular procedure performance in residents [4-6], and this system provides an opportunity to perform endovascular procedures in a safe environment as an educational tool for novice residents.

However, due to the late entry of simulation systems in China, there has been no report of the use of 3-dimensional vascular simulation systems in clinical practice teaching in the area of vascular surgery. Therefore, we have retrospectively collected the teaching data of residents who received the simulation training and those who did

For peer review only - http://bmjopen.bmj.com/site/about/guidelines.xhtml

not in our hospital. The aim of this study was to assess the effect of simulation-based training on improving the technical performance and subsequent clinical procedures of residents in vascular surgery.

METHODS

Study procedures

A total of 95 vascular resident trainees at the First Affiliated Hospital of Xi'an Jiaotong University were respectively collected in this study from Jan 2015 to Dec 2018, 47 vascular residents received simulation-based vascular training (ST group) for two weeks, and then they completed the last clinical training. The other 48 residents only completed conventional clinical training (CT group, including classroom teaching, lectures and surgical practice) without the simulation course, and all residents needed to complete 6 months of endovascular training in vascular surgery. A survey was administered to determine the demographics, academic degree, specialty level, and previous work experiences that may have been relevant in terms of the residents' ability to learn vascular interventional skills. This study was approved by the institutional review and ethics board of the First Affiliated Hospital of Xi'an Jiaotong University (XJTU1AF2014LSK-112), all of the patients provided written informed consent.

Before the course was performed, the residents in the ST group received a standardized introduction to the endovascular simulator and performed a cerebrovascular angiography procedure. The 2-week curriculum consisted of theory teaching and 30-60 min lectures per day covering basic catheter-based interventions, cerebrovascular disease, superficial femoral artery disease and renal artery disease. The

For peer review only - http://bmjopen.bmj.com/site/about/guidelines.xhtml

residents received 1-hour mentored simulator sessions per day and practiced carotid, renal and superficial femoral artery interventions. This course was performed by a tutor. Then, residents needed to complete the primary endovascular procedure with direct instruction. During the entire training process, each resident was required to complete the simulation training for no less than 1 hour per day. The course concluded with a final cerebrovascular angiography procedure performed on the simulator. The residents in the CT group underwent the basic 2-week curriculum consisting of theory teaching and 30-60 min lectures per day covering basic endovascular intervention procedures but without the simulation training course.

Simulation system

The vascular simulator system (Angiomentor system, Simbionix, Ltd., Cleveland, OH, Fig 1) was composed of a standard desktop computer with software that simulated the human arterial system in 3 dimensions, and any user could perform the endovascular interventional procedures under the instruction of the system. This simulation system was connected to a haptic pressure feedback module, which used a force feedback system to detect external devices. When the users inserted the angiography catheters and wires, injected contrast and performed the endovascular procedures with digital subtraction angiography, all procedures could then be displayed on the screen in real time. The user was able to select the device to be simulated through one monitor, and the second monitor was used to display the simulated fluoroscopic image.

Procedure evaluation

When the residents completed the training course, all residents received assessments of

For peer review only - http://bmjopen.bmj.com/site/about/guidelines.xhtml

theoretical knowledge at 1 month and practical assessments at 6 months. The teaching expert group made the theoretical test questions based on the key points of vascular disease as well as the endovascular interventional procedures and technical points involved in vascular surgery clinical training (the theoretical knowledge test is formulated according to the standardized question bank and the simulation system question bank), and then the residents completed the assessment independently. Finally, the expert group determined the student's score based on the results of the test (including the detailed information of knowledge and practice). The theoretical score range was 0-10, and a passing score was defined as a score higher than 7. All residents completed the arterial puncture procedure with the Seldinger technique and performed the cerebrovascular angiography procedure. The success criterion of arterial puncture was defined as follows: all puncture procedures were successfully completed, and the sheath was successfully inserted into the femoral artery. If the residents failed to complete the processes of puncture and sheath placement, which was defined as a puncture failure, then the puncture was performed by the teacher. The puncture success rate, puncture time and complications were recorded, and the comfort scores of the patients during the puncture procedure were assessed with a numerical rating scale (NRS). The NRS score ranged from 0 to 10, where 0 indicates worst uncomfortable pain and 10 indicates comfortable pain [7]. Subsequently, the residents needed to complete the cerebrovascular angiography procedure. The evaluation indicators of angiography were as following: learn time to complete the procedure (LTP, defined as the time from the beginning of training to the completion of the first angiography

For peer review only - http://bmjopen.bmj.com/site/about/guidelines.xhtml

independently), time of complete procedure at the final test (TCP), fluo time of procedure (FTP), cumulative air kerma (CAK) and dose area product (DAP). TCP, FTP, CAK and DAP were defined as the time of procedure and related parameters of the assessed angiography procedure at the end of the training.

Patient and public involvement

Patients and the public were not involved in the design or planning of the study.

Statistical Analysis

All data were analyzed using SPSS v. 11.0 (SPSS, Chicago, IL, USA), and $P < .05$ was considered statistically significant. To test the difference between groups, we used Chi-square analysis for categorical variables and Student's t-tests for continuous variables, and we tested the significance of the difference between 2 independent proportions when the results were presented as percentages.

RESULTS

The baseline data of the two groups

This study included 48 residents and 47 residents who were retrospectively recruited in this study, and there was no significant difference in baseline data between the two groups (Table 1, $P>0.05$). There were 44 males and 4 females who received CT training, with a mean age of 33.13 years, and 42 males and 5 females who received ST training, with a mean age of 33.91 years ($P>0.05$). The background academic degrees were bachelor and postgraduate degrees in the CT and ST groups ($P>0.05$), and 30 and 26 residents had a specialty background in vascular surgery in both groups (62.50% vs 55.32%, $P>0.05$). The clinical work experience of most residents was less than three

For peer review only - http://bmjopen.bmj.com/site/about/guidelines.xhtml

years in both groups (66.67% vs 57.45, P>0.05).

The theoretical scores between both groups

All residents passed the training test; however, the mean scores of the residents were higher in the ST group than in the CT group (8.74±1.09 vs 8.13±1.31, P=0.014). After the clinical training, the training satisfaction rates of all residents were similar (97.87% vs 91.67%, P>0.05); however, when asked whether they wished to participate in the training again, the residents in the ST group showed a higher willingness rate than residents in the CT groups (93.62% vs 79.17%, P=0.04).

The performance of arterial puncture in residents

After the training, all residents underwent an arterial puncture test (Table 2), and the success rate of the procedure was higher in the ST group than in the CT group (78.72% vs 58.33%, P=0.033); however, the total puncture success rate was similar between the two groups (95.74% vs 91.67%, P>0.05). The complications of the puncture sites included bruising, hematoma, infection and pseudoaneurysm, and there was no significant difference in the incidence of complications between the ST and CT groups (17.02% vs 18.75%, P>0.05); however, the time of the puncture procedure was shorter in the ST group than in the CT group (9.56±5.24 min vs 12.15±6.87 min, P=0.002), and the comfort score of patients was higher in the ST group than in the CT group according to the NRS scores (5.49±1.72 vs 4.71±1.57, P=0.023).

The outcome of cerebrovascular angiography

All residents needed to complete the final cerebrovascular angiography test; the related parameters are listed in Table 3. The residents in the ST group showed a shorter study

For peer review only - http://bmjopen.bmj.com/site/about/guidelines.xhtml

curve with a lower mean LTP than residents in the CT groups (3.65 mon vs 4.07 mon, $P<0.01$), and the TCP of the final test was higher in the CT group than in the ST group (41.32 min vs 33.81 min, $P=0.002$). The radiation damage-related parameters were recorded, and the residents in the CT group showed higher mean values of FTP (631.47 s vs 489.33 s, $P<0.001$) and CAK than the residents in the ST group (463.16 vs 401.30 mGy, $P=0.043$); however, the mean DAP value in both groups indicated no difference (128624.30 mGy.cm^2 vs 128012.10 mGy.cm^2, $P>0.052$).

DISCUSSION

The development of vascular surgery occurred relatively late in China; thus, the training model used for vascular residents in most university hospitals was traditional training; however, traditional training did not improve residents' understanding and interest in vascular surgery. In past decades, there were fewer residents who chose vascular surgery as a career option in China, which was consistent with the reports of previous studies [7]. Therefore, improving residents' interest in vascular surgery and promoting vascular clinical performance have been the main problems associated with clinical training [8]. Earlier studies have shown that compared with traditional training, the use of network media, social media and simulation systems can achieve better training results [9-11]. In this study, we used the vascular simulation system to assist residents in clinical training. The results revealed that simulation training could significantly improve the clinical practice effect of residents. The residents who received the simulation training had significantly higher theoretical scores; in addition, the interest level of residents was higher after simulation training. The vascular simulation system

For peer review only - http://bmjopen.bmj.com/site/about/guidelines.xhtml

For peer review only

could standardize complex vascular systems and different vascular lesions through analog calculations, which could help residents practice vascular skills training earlier and improve the interest and performance of residents [3]. Our result reveals that the residents could deeply understand theoretical knowledge and practical knowledge through simulation training, which also helps to improve the residents' theoretical and practical knowledge.

After simulation training and basic training, residents need further vascular operation training under the guidance of tutors, and the basic procedure for vascular residents is arterial puncture. However, it is impossible for residents to repeatedly perform the procedure during the treatment of patients; thus, in vitro simulation training has become the main teaching method in the training of vascular residents. Residents who received simulator training showed better clinical performance in the vascular surgery rotation, more motivation to learn, a shorter learning time and a lower number of clinical procedural errors, and the patients indicated a lower discomfort rate with the procedure [12, 13]. In our study, the results confirmed that the residents who underwent the simulation training had a significantly higher success rate of arterial puncture, and the time of the puncture procedure was also significantly lower. Each step of the vascular procedure could be programmed and standardized in the simulation system. After the teacher's explanation and auxiliary training, it was easier for residents to develop standardized vascular skill habits and the correct procedural process. Finally, we evaluated the training effect with the cerebrovascular angiography procedure. Our results proved that the learning time of the angiography procedure and time of

For peer review only - http://bmjopen.bmj.com/site/about/guidelines.xhtml

completed procedures were significantly lower in residents with simulation training; these results were consistent with previous reports [14]. This finding also confirmed that different simulation systems and teaching models could improve the effectiveness of clinical teaching and promote the understanding and proficiency of vascular practical performance.

Furthermore, the final effect of clinical training should be assessed by the practice procedure of residents. Our study confirmed that vascular simulation training could promote the practice knowledge of residents and promote the understanding of vascular surgical procedure. Most cases of vascular disease teaching need to be performed under radiation; thus, simulation training could avoid radiation damage to teachers, patients and residents. In this study, the results demonstrated that simulation training could decrease the fluo time and cumulative air kerma of the procedure, which meant that simulation training could reduce the radiation damage of patients and residents, and radiation protection was an important teaching ethics component in vascular surgery practice. However, due to the limitations of our teaching funding and the time course, only selective residents underwent the simulation training for 2 weeks, which was different from what occurs in advanced vascular centers. Other reports have shown that simulation training for 8 weeks can improve the procedure skills of residents, contribute to patient safety and have a positive impact on the career planning and choice of vascular surgeons [15]. Therefore, the simulation training should be the basic course for residents. Therefore, the simulation training should be the basic course for residents, especially in pre-career students and residents in rotation, meanwhile the simulation

For peer review only - http://bmjopen.bmj.com/site/about/guidelines.xhtml

training should be well-defined and step-planned, otherwise, the simulation training may result in an impaired learning and worse performance in real clinical practice [16].

Limitations

There were several limitations. First, this study was not a prospective randomized study, and the conclusions of this study should be confirmed in the future; Second, when compared with Western counties, the simulation training is not wildly used in China, thus the truly effect of the simulation training is not investigated deeply; Third, the clinical performance of the residents should be evaluated via the real clinical procedure after the simulation training, this is the next step of our study.

Conclusions

Our results confirmed that a vascular simulation system could improve the clinical skills of residents and reduce the radiation damage received by patients and residents in endovascular procedures.

Author Contributions: LY, YZ L: data collection. LY, YZ L, JL L and YM L: conception or design of the work; data analysis and interpretation; critical revision of the article; drafting of the article and final approval of the version to be published.

Acknowledgments: The language of this article was edited by American Journal Experts (AJE). Thanks to Prof. Jian Yang from the clinical research center of the First Affiliated Hospital of Xi'an Jiaotong University for providing statistical analysis support and proofread.

Funding: This project was supported by the Natural Science Foundation of China (NO.8197 0365)

For peer review only

For peer review only - http://bmjopen.bmj.com/site/about/guidelines.xhtml

Conflicts of Interest: None.

Patient consent for publication: Not required.

Ethical approval: This study was approved by the institutional review and ethics board of the First Affiliated Hospital of Xi'an Jiaotong University.

Data availability statement: Data are available upon reasonable request.

For peer review only

For peer review only - http://bmjopen.bmj.com/site/about/guidelines.xhtml

References:

[1] Song P, Rudan D, Wang M, Chang X, Rudan I. National and subnational estimation of the prevalence of peripheral artery disease (PAD) in China: a systematic review and meta-analysis. J Glob Health. 2019;9010601.

[2] Mills J. Vascular surgery training in the United States: A half-century of evolution. J Vasc Surg. 2008;48:90S-97S.

[3] Lee JT, Qiu M, Teshome M. The utility of endovascular simulation to improve technical performance and stimulate continued interest of preclinical medical students in vascular surgery. J Surg Educ, 2010, 66: 367-369.

[4] Aggarwal R, Black S, Hance J, et al. Virtual reality simulation training can improve inexperienced surgeons' endovascular skills. Eur J Vasc Endovasc Surg. 2007;31:588-593.

[5] Chaer RA, Derubertis BG, Lin SC, et al. Simulation improves resident performance in catheter-based intervention: Results of a randomized, controlled study. Ann Surg. 2006;244:343-352.

[6] Tedesco MM, Pak JJ, Harris EJ, et al. Simulation-based endovascular skills assessment: The future of credentialing? J Vasc Surg. 2008;47:1008-1001.

[7] Farrar JT, Young JP Jr, LaMoreaux L, Werth JL, Poole RM. Clinical importance of changes in chronic pain intensity measured on an 11-point numerical pain rating scale. Pain 2001; 94:149–158.

[8] Zimmerman P, O'Brien-Irr M, Dryjski M, et al. The new training paradigms and the unfilled match positions of 2004: Will history repeat itself? J Vasc Surg. 2006;

For peer review only - http://bmjopen.bmj.com/site/about/guidelines.xhtml

44:145-150.

[9] Seymour N, Gallager A, Roman S, et al. Virtual reality training improves operating room performance: Results of a randomized, double-blind study. Ann Surg. 2002;236:458-463.

[10] Raval MV, Wang X, Cohen ME, et al. The influence of resident involvement on surgical outcomes. J Am Coll Surg. 2011;212:889-898.

[11] Smith BK, Greenberg JA, Mitchell EL. The evolving integrated vascular surgery residency curriculum. Ann Vasc Surg. 2014;28:1761-1768.

[12] Van Sickle K, Ritter E, Baghai M, et al. Prospective, randomized, double-blind trial of curriculum-based training for intracorporeal suturing and knot-tying. J Am Coll Surg. 2008;207: 560-568.

[13] Tan SS, Sarker SK. Simulation in surgery: a review. Scott Med J. 2011;56:104-109.

[14] Sturm LP, Windsor JA, Cosman PH, Cregan P, Hewett PJ, Maddern GJ. A systematic review of skills transfer after surgical simulation training. Ann Surg. 2008;248:166-179.

[15] Egle JP, Subramanyam Malladi SV, Gopinath N, Mittal VK. Simulation Training Improves Resident Performance in Hand-Sewn Vascular and Bowel Anastomoses. J Surg. 2014;72:291-296.

[16] Jensen UJ, Jensen J, Olivecrona G, Ahlberg G, Lagerquist B, Tornvall P. The role of a simulator-based course in coronary angiography on performance in real life cath lab. BMC Med Educ. 2014;14:49.

For peer review only - http://bmjopen.bmj.com/site/about/guidelines.xhtml

Figure legends

Figure 1. The vascular simulator system (Angiomentor system, Simbionix, Ltd., Cleveland, OH) used in this study simulated the vascular interventional procedures: a) work station; b) pedal; c) simulator.

For peer review only

For peer review only - http://bmjopen.bmj.com/site/about/guidelines.xhtml

Tables

Table 1 The baseline data of both groups

	CT group (n=48)	ST group (n=47)	*P* value
Sex (M)	44 (91.67)	42 (89.36)	0.70
Age (years)	33.13±3.04	33.91±4.94	0.35
Academic degree			0.36
bachelor	22 (45.83)	26 (55.32)	
postgraduate	26 (54.17)	21 (44.68)	
Specialty background			0.48
vascular	30 (62.50)	26 (55.32)	
nonvascular	18 (37.50)	21 (44.68)	
Work experience			0.35
> 3 years	16 (33.33)	20 (42.55)	
≤ 3 years	32 (66.67)	27 (57.45)	

CT: conventional training; ST: simulation training; M: male.

P value, compared with the CT group

For peer review only

For peer review only - http://bmjopen.bmj.com/site/about/guidelines.xhtml

407

408 **Table 2 The performance of arterial puncture in residents**

	CT group (n=48)	ST group (n=47)	*P* value
Puncture success rate	28 (58.33)	37 (78.72)	0.033
Total puncture success rate	44 (91.67)	45 (95.74)	0.69
Complications from puncture	9 (18.75)	8 (17.02)	0.83
bruising	5 (10.42)	6 (12.77)	
hematoma	3 (6.25)	2 (4.26)	
infection	0	0	
arteriovenous fistula	0	0	
pseudoaneurysm	1 (2.08)	0	
time of puncture (min)	12.15±6.87	9.56±5.24	0.002
Comfort score of patients	4.71±1.57	5.49±1.72	0.023

409 CT: conventional training; ST: simulation training.

410 *P* value, compared with the CT group

411

412

413

414

415

416

417

For peer review only - http://bmjopen.bmj.com/site/about/guidelines.xhtml

Table 3 The performance on cerebrovascular angiography in residents

	CT group (n=48)	ST group (n=47)	*P* value
LTP (mon)	4.07±0.77	3.65±0.64	<0.01
TCP (min)	41.32±12.56	33.81±10.11	0.002
FTP (s)	631.47±243.65	489.33±237.13	0.005
CAK (mGy)	463.16±134.14	401.30±149.06	0.043
DAP (mGy.cm^2)	128624.30±28982.22	128012.10±31035.08	0.92

CT: conventional training; ST: simulation training; LTP: learn time to complete procedure from beginning; TCP: time of complete procedure at the final test; FTP: fluo time of procedure; CAK: cumulative air kerma; DAP: dose area product

P value, compared with the CT group

For peer review only

For peer review only - http://bmjopen.bmj.com/site/about/guidelines.xhtml

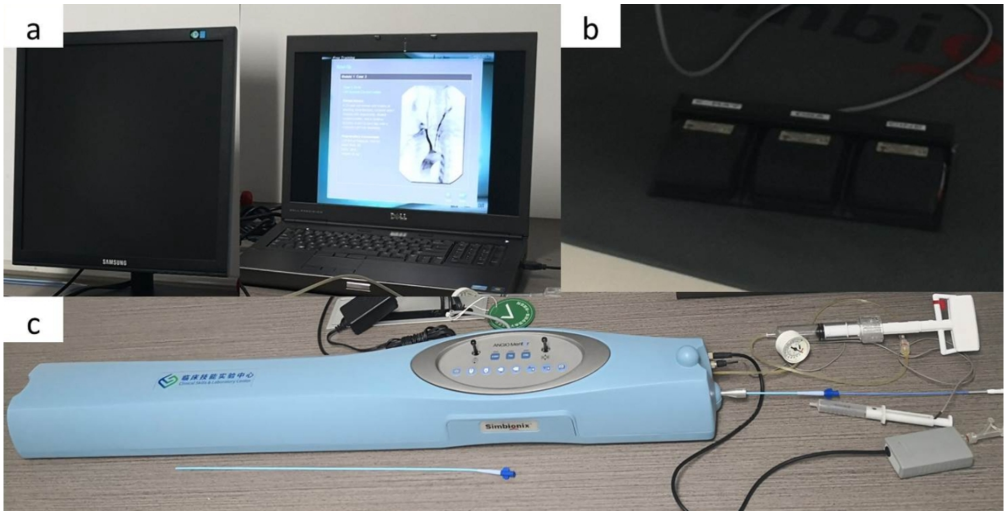

Figure 1. The vascular simulator system (Angiomentor system, Simbionix, Ltd., Cleveland, OH) used in this study simulated the vascular interventional procedures: a) work station; b) pedal; c) simulator.

124x63mm (500 x 500 DPI)

STROBE 2007 (v4) checklist of items to be included in reports of observational studies in epidemiology*
Checklist for cohort, case-control, and cross-sectional studies (combined)

Section/Topic	Item #	Recommendation	Reported on page #
**Title and abstract**	1	(*a*) Indicate the study's design with a commonly used term in the title or the abstract	1–2
		(*b*) Provide in the abstract an informative and balanced summary of what was done and what was found	1–2
**Introduction**			
Background/rationale	2	Explain the scientific background and rationale for the investigation being reported	3–4
Objectives	3	State specific objectives, including any pre-specified hypotheses	3–4
**Methods**			
Study design	4	Present key elements of study design early in the paper	4–6
Setting	5	Describe the setting, locations, and relevant dates, including periods of recruitment, exposure, follow-up, and data collection	4–6
Participants	6	(*a*) *Cohort study*—Give the eligibility criteria, and the sources and methods of selection of participants. Describe methods of follow-up<br>*Case-control study*—Give the eligibility criteria, and the sources and methods of case ascertainment and control selection. Give the rationale for the choice of cases and controls<br>*Cross-sectional study*—Give the eligibility criteria, and the sources and methods of selection of participants	4–6
		(*b*) *Cohort study*—For matched studies, give matching criteria and number of exposed and unexposed<br>*Case-control study*—For matched studies, give matching criteria and the number of controls per case	4–6
Variables	7	Clearly define all outcomes, exposures, predictors, potential confounders, and effect modifiers. Give diagnostic criteria, if applicable	4–6
Data sources/ measurement	8*	For each variable of interest, give sources of data and details of methods of assessment (measurement). Describe comparability of assessment methods if there is more than one group	4–6
Bias	9	Describe any efforts to address potential sources of bias	4–6
Study size	10	Explain how the study size was arrived at	4–6
Quantitative variables	11	Explain how quantitative variables were handled in the analyses. If applicable, describe which groupings were chosen and why	4–6
Statistical methods	12	(*a*) Describe all statistical methods, including those used to control for confounding	7
		(*b*) Describe any methods used to examine subgroups and interactions	7
		(*c*) Explain how missing data were addressed	7
		(*d*) *Cohort study*—If applicable, explain how loss to follow-up was addressed<br>*Case-control study*—If applicable, explain how matching of cases and controls was addressed	7

For peer review only

		Cross-sectional study—If applicable, describe analytical methods taking account of sampling strategy	
		(*e*) Describe any sensitivity analyses	7
**Results**			
Participants	13*	(a) Report numbers of individuals at each stage of study—eg numbers potentially eligible, examined for eligibility, confirmed eligible, included in the study, completing follow-up, and analysed	7–9
		(b) Give reasons for non-participation at each stage	7–9
		(c) Consider use of a flow diagram	7–9
Descriptive data	14*	(a) Give characteristics of study participants (eg demographic, clinical, social) and information on exposures and potential confounders	7–9
		(b) Indicate number of participants with missing data for each variable of interest	7–9
		(c) *Cohort study*—Summarise follow-up time (eg, average and total amount)	7–9
Outcome data	15*	*Cohort study*—Report numbers of outcome events or summary measures over time	7–9
		Case-control study—Report numbers in each exposure category, or summary measures of exposure	7–9
		Cross-sectional study—Report numbers of outcome events or summary measures	7–9
Main results	16	(*a*) Give unadjusted estimates and, if applicable, confounder-adjusted estimates and their precision (eg, 95% confidence interval). Make clear which confounders were adjusted for and why they were included	7–9
		(*b*) Report category boundaries when continuous variables were categorized	7–9
		(*c*) If relevant, consider translating estimates of relative risk into absolute risk for a meaningful time period	7–9
Other analyses	17	Report other analyses done—eg analyses of subgroups and interactions, and sensitivity analyses	7–9
**Discussion**			
Key results	18	Summarise key results with reference to study objectives	9–11
Limitations	19	Discuss limitations of the study, taking into account sources of potential bias or imprecision. Discuss both direction and magnitude of any potential bias	9–11
Interpretation	20	Give a cautious overall interpretation of results considering objectives, limitations, multiplicity of analyses, results from similar studies, and other relevant evidence	9–11
Generalisability	21	Discuss the generalisability (external validity) of the study results	9–11
**Other information**			
Funding	22	Give the source of funding and the role of the funders for the present study and, if applicable, for the original study on which the present article is based	11

*Give information separately for cases and controls in case-control studies and, if applicable, for exposed and unexposed groups in cohort and cross-sectional studies.

Note: An Explanation and Elaboration article discusses each checklist item and gives methodological background and published examples of transparent reporting. The STROBE checklist is best used in conjunction with this article (freely available on the Web sites of PLoS Medicine at http://www.plosmedicine.org/, Annals of Internal Medicine at http://www.annals.org/, and Epidemiology at http://www.epidem.com/). Information on the STROBE Initiative is available at www.strobe-statement.org.

For peer review only
